# Seafood Contamination after the BP Gulf Oil Spill and Risks to Vulnerable Populations: A Critique of the FDA Risk Assessment

**DOI:** 10.1289/ehp.1103695

**Published:** 2011-10-12

**Authors:** Miriam Rotkin-Ellman, Karen K. Wong, Gina M. Solomon

**Affiliations:** 1 Natural Resources Defense Council, San Francisco, California, USA; 2 Department of Medicine, University of California San Francisco, San Francisco, California, USA

**Keywords:** BP oil spill, children’s health, *Deepwater Horizon*, Food and Drug Administration, Gulf of Mexico, PAHs, polycyclic aromatic hydrocarbons, risk assessment, seafood

## Abstract

Background: The BP oil spill of 2010 resulted in contamination of one of the most productive fisheries in the United States by polycyclic aromatic hydrocarbons (PAHs). PAHs, which can accumulate in seafood, are known carcinogens and developmental toxicants. In response to the oil spill, the U.S. Food and Drug Administration (FDA) developed risk criteria and established thresholds for allowable levels [levels of concern (LOCs)] of PAH contaminants in Gulf Coast seafood.

Objectives: We evaluated the degree to which the FDA’s risk criteria adequately protect vulnerable Gulf Coast populations from cancer risk associated with PAHs in seafood.

Discussion: The FDA LOCs significantly underestimate risk from seafood contaminants among sensitive Gulf Coast populations by failing to *a*) account for the increased vulnerability of the developing fetus and child; *b*) use appropriate seafood consumption rates; *c*) include all relevant health end points; and *d*) incorporate health-protective estimates of exposure duration and acceptable risk. For benzo[*a*]pyrene and naphthalene, revised LOCs are between two and four orders of magnitude below the level set by the FDA. Comparison of measured levels of PAHs in Gulf seafood with the revised LOCs revealed that up to 53% of Gulf shrimp samples were above LOCs for pregnant women who are high-end seafood consumers.

Conclusions: FDA risk assessment methods should be updated to better reflect current risk assessment practices and to protect vulnerable populations such as pregnant women and children.

The Gulf of Mexico is a very productive fishery, comprising the majority of domestic shrimp (60%) and oyster (70%) production (Louisiana Seafood Promotion & Marketing Board 2010). During the BP *Deepwater Horizon* oil spill, > 200 million gallons of oil poured into the Gulf of Mexico, followed by 1.8 million gallons of dispersants intended to break down the oil into droplets (Repanich 2010).

The U.S. Food and Drug Administration (FDA) is the agency responsible for determining seafood safety. In response to the oil spill, the FDA, working with the states and the National Oceanic and Atmospheric Administration (NOAA), initially closed approximately 37% of the Gulf of Mexico (225,290 km^2^) to commercial and recreational fishing (NOAA 2010). Reopening of these areas was conducted on a rolling basis, using a two-phase testing regime consisting of organoleptic testing, in which experts sniff pieces of seafood for oil taint, and chemical analysis for polycyclic aromatic hydrocarbons (PAHs) (FDA 2010a). PAHs are found in crude oil and have the potential to accumulate in aquatic organisms, presenting a health risk via ingestion of contaminated seafood (Yender et al. 2002). Crustaceans and mollusks, such as shrimp, crab, and oysters, are especially likely to be contaminated because of reduced rates of biological clearance of PAHs in these species (Law et al. 2002). The FDA tested for the presence of 13 PAHs selected on the basis of known carcinogenicity or other health effects, including stunted growth, anemia, and kidney disease. The FDA also calculated allowable thresholds [levels of concern (LOCs)] for PAHs in each specific type of Gulf seafood.

The FDA allowed most Gulf fisheries to reopen during the summer and fall of 2010 based on measured PAHs in seafood below the LOCs, although public confidence in Gulf seafood was slow to rebuild (Marcus 2011). The adequacy of the policy decision to resume commercial fishing hinged on the accuracy of FDA’s assumptions in calculating the LOCs and on the rigor of the seafood monitoring program. By critically evaluating the FDA’s risk assessment and monitoring practices, we aimed to determine the adequacy of public health protection in this particular case and to identify any broader improvements that may be needed to risk assessment practices and food safety determinations at the FDA.

## Objectives

We evaluated the degree to which the FDA’s procedures for determining the safety of Gulf seafood after the BP oil spill (FDA 2010a) reflect current risk assessment practices and protect vulnerable populations. We focused on cancer risk associated with shellfish consumption, calculated revised LOCs designed to be protective of vulnerable populations, and compared them with the FDA LOCs as well as with measured concentrations of PAHs in Gulf shellfish.

## Discussion

The FDA Gulf seafood risk assessment (FDA 2010a) contains numerous assumptions that are inconsistent with the FDA’s own prior practice and with risk assessment guidelines produced by other authoritative entities, including the National Research Council (NRC), the World Health Organization (WHO), the U.S. Environmental Protection Agency (EPA), and the California EPA. Each of these assumptions would tend to result in an underestimate of risk for a significant fraction of the exposed population. The questionable assumptions include six main issues: *a*) high consumer body weight, *b*) low estimates of seafood consumption, *c*) failure to include a cancer risk assessment for naphthalene, *d*) failure to adjust for early-life susceptibility to PAHs, *e*) short exposure duration, and *f* ) high cancer risk benchmarks. Taken together, these flaws illustrate a failure to incorporate the substantial body of evidence on the increased vulnerability of subpopulations to contaminants, such as PAHs, in seafood.

*High consumer body weight.* For derivation of all LOCs, the FDA assumed a body weight of 80 kg (176 lb). Although the FDA’s body weight assumption is reasonable for some segments of the population, close to 75% of the female population in the United States weighs < 80 kg, and the average body weight of a 4- to 6-year-old child is 21.6 kg (McDowell et al. 2008). In a follow-up risk assessment conducted for an additional oil spill–related contaminant, the FDA acknowledged that using a lower body weight (60 kg) offered greater health protection (Bolger 2010). The U.S. EPA publishes age group–specific body weights for use in risk assessments (U.S. EPA 2011), based on the broad scientific understanding that children have increased susceptibility to ingested contaminants because of their high food intake in proportion of their body weight (NRC 1993). Because acceptable intake of contaminants is calculated as a fraction of body weight, using an inflated assumption in a risk assessment is systematically underprotective of the entire population that weighs below the level used in the calculation.

*Low estimate of seafood consumption.* The FDA assumed that each consumer eats a daily average of 49, 12, or 13 g of fish, oysters, or shrimp/crab, respectively. The FDA derived this consumption rate from the 90th percentile reported in the 2005–2006 National Health and Nutrition Examination Survey (NHANES) for nationwide seafood consumption (FDA 2010a). Populations living along the Gulf Coast have a rate of seafood consumption higher than the rest of the nation (Mahaffey et al. 2009). For example, surveys of New Orleans, Louisiana, residents and recreational anglers in Louisiana found high-end consumers reporting shrimp intakes of 65.1 and 55.5 g/day, respectively (Anderson and Rice 1993; Lincoln et al. 2011), which is significantly higher than the FDA’s estimate of 13 g/day. Federal and international agencies, including the U.S. EPA and the WHO, have identified the need to protect high-end and subsistence fishing communities from contaminants in seafood by accounting for increased consumption rates. These agencies recommend using local studies and/or the 95th–97th percentile of national consumption surveys (U.S. EPA 2000; WHO 2008), in contrast to the 90th percentile used by the FDA. To protect subsistence adult consumers, the U.S. EPA recommends fish consumption rates ranging from 142.4 g/day (general population) to 170 g/day (Native Americans) (U.S. EPA 2000), which is 2.9–3.5 times higher than the FDA estimate of 49 g/day. Similarly, the 95th percentile fish consumption rate reported in *Seafood Choices: Balancing Benefits and Risks* (Institute of Medicine 2007) is equal to 155 g/day—3.2 times higher than the FDA assumption. The FDA also failed to account for the possibility that consumers may eat a combination of various types of seafood when they calculated consumption rates and LOCs for shrimp, oysters, crab, and fish separately.

*Failure to consider the cancer risk from naphthalene.* Naphthalene was one of the most frequently detected PAHs in Gulf seafood tested after the spill and was the most prevalent PAH in the oil itself (FDA 2010a; FDA, unpublished data). Despite the fact that naphthalene poses a health risk due to both carcinogenic and noncarcinogenic health effects, the FDA established the LOC in Gulf seafood based solely on noncancer effects (FDA 2010a). Naphthalene is listed in the *12th Report on Carcinogens* [National Toxicology Program (NTP) 2011] (which the FDA has endorsed) as reasonably anticipated to be a human carcinogen based on dose-related rare nasal and respiratory neuroblastomas and adenomas in male and female rats, and on lung tumors in female mice. Inhalation has been associated with cancer of the larynx in humans, and ingestion was associated with human colorectal cancer in one study (NTP 2011). Naphthalene is also listed by the State of California as known to cause cancer, with sufficient evidence to determine a cancer potency factor of 0.12 per mg/kg-day, which defines the relationship between exposures and cancer risk [Office of Environmental Health Hazard Assessment (OEHHA) 2005].

The FDA did not assess whether exposures in Gulf seafood could pose an increased risk of cancer from naphthalene. Because PAHs are a mixture of multiple compounds, small exposures to multiple PAHs can add up to significant cancer risks. By omitting naphthalene from its cancer risk assessment, the FDA ignored the potential cumulative effect of exposures to multiple carcinogens.

*Failure to include early-life vulnerability.* The FDA conducted a single risk assessment for adults and did not evaluate potential increased risks to the developing fetus or child, yet exposure to PAHs during pregnancy causes genetic damage to the developing fetus (Harper et al. 1989; Orjuela et al. 2010). Most PAHs are lipid soluble and therefore cross the placenta (Calabrese 1978; Shendrikova and Aleksandrov 1974). PAHs have also been observed in human breast milk (Del Bubba et al. 2005; Kim et al. 2008). Animal studies have found that ingestion of PAHs during pregnancy results in much greater genetic damage in the fetus than in the mother (Harper et al. 1989). Children exposed prenatally to PAHs have statistically significant increases in DNA aberrations in specific chromosomes, low birth weight, and intrauterine growth restriction (Choi et al. 2006; Dejmek et al. 2000; Orjuela et al. 2010; Perera et al. 2003, 2005).

The increased vulnerability of the developing fetus and child to genotoxins and carcinogens has been widely recognized. In March 2005, the U.S. EPA released the *Supplemental Guidance for Assessing Susceptibility from Early-Life Exposure to Carcinogens* (U.S. EPA 2005), which presented age-dependent adjustment factors (ADAFs). ADAFs adjust the slope factors to account for differences in carcinogen potency by age groups, based on data from animal studies of cancer potency in early-life stages compared with adult animals (U.S. EPA 2005). The U.S. EPA methods also use different rates of exposure according to age, accounting for the relative difference in intake between children and adults. The U.S. EPA did not include ADAFs for prenatal exposures, but did acknowledge that the available data support increased prenatal susceptibility (U.S. EPA 2005). In California, the OEHHA, under the California EPA, accounts for childhood exposures in its risk assessment methods and provides an adjustment factor [age sensitivity factor (ASF)] for prenatal exposures (OEHHA 2009). The FDA did not incorporate any of this information into its calculation of the LOCs.

*Short exposure duration and less-protective cancer risk benchmarks.* The FDA LOC incorporates a duration of exposure of only 5 years and an acceptable rate of cancer of 1 cancer in 100,000 people. However, based on prior experience from oil spills, PAHs are detectable in shellfish for up to 13 years after oil contamination, and there is evidence of ongoing DNA damage from PAHs in marine life after that time (Bejarano and Michel 2010; Thomas et al. 2007). There is considerable variation in the half-life of PAHs, depending on the structure of the compound and environmental conditions. However, using an average value recommended by the California EPA for PAHs in soil (570 days), approximately 10% of the contamination would be expected to remain after 5 years, and < 2% would remain after 10 years (OEHHA 2000). FDA risk assessments conducted for prior oil spills, such as the *Exxon Valdez*, used more conservative and health-protective values for these parameters: a 10-year exposure duration and an acceptable cancer risk level of 1 in 1 million (Bolger and Carrington 1999).

*Revised risk assessment and LOCs.* We used published sources to estimate exposure scenarios for three populations vulnerable to PAH contamination in Gulf Coast seafood: a woman (or small man), a pregnant woman (prenatal exposure to < 10 years of age), and a child (2–12 years of age). See Table 1 and Supplemental Material, pp. 2–3 (http://dx.doi.org/10.1289/ehp.1103695) for a description and comparison of the exposure and risk profiles. Using the vulnerable population risk profiles, the FDA LOC equation (adult scenario), and the U.S. EPA/California EPA ADAFs/ASFs and risk calculation methods (child and pregnant woman scenarios), we derived revised LOCs for benzo[*a*]pyrene (BaP), one of the most potent PAHs, and for cancer risk from naphthalene in individual types of seafood and for a combined cumulative shellfish-rich diet. Consistent with FDA methods, we used toxic equivalencies to translate the LOC for BaP to other (non-naphthalene) carcinogenic PAHs detected in seafood (see Supplemental Material, Table 1).

**Table 1:** Parameters to estimate cancer risk due to PAHs in Gulf seafood: FDA versus vulnerable-populations method.

Vulnerable populations								
FDA	Pregnant woman*a*	Infant	Child*b*		
Risk scenario	Adult	Woman	Prenatal	0 to < 2	2 to 5	6 to < 12
Acceptable risk level		1 in 100,000		1 in 1,000,000*c*								
Exposure duration (years)		5		10*c*								
Body weight (kg)		80		60*d *		60*e *		9.6*f *		17.4*f *		31.8*f*
Consumption rate (g/day)												
Fish		49		155*g *		15.5*h *		16.9*i *		84.4*g*		86.4*g*
Shrimp		13		44*g *		4.4*h *		10.1*j *		24.0*g*		24.6*g*
Crab		13		21*g *		2.1*h *		4.7*j *		11.2*g*		11.5*g *
Oysters		12		18*k *		1.8*h *		2.4*l *		5.8*l *		10.7*l *
Early-life vulnerability adjustment												
Child*m *		None		NA		NA		NA		3		3
Pregnant woman/infant/child*n *		None		NA		3		13		5		3
NA, not applicable. **a**Pregnant woman scenario, third trimester to 9.75 years of age. **b**Child scenario, 2 to < 12 years of age. **c**Value used in *Exxon Valdez* risk assessment (Bolger and Carrington 1999). **d**Value used in FDA risk assessment for dispersant chemicals in Gulf seafood (Bolger 2010). **e**Prenatal dose calculated based on woman’s body weight per OEHHA (2009). **f**Data from U.S. EPA (2008). **g**95th percentile from IOM (2007). **h**Fetal PAH exposure assumed to be 10% of maternal exposure based on animal dose studies (Perera et al. 2005). **i**Data from U.S. EPA (2008), high-end fish consumers. **j**Estimated using the U.S. EPA CSEF early-life total fish consumption distribution and consumption rates for 2- to 5-year-old children [see Supplemental Material, p. 3 (http://dx.doi.org/10.1289/ehp.1103695)]. **k**Data from Louisiana anglers study (Lincoln et al. 2011). **l**Data from U.S. EPA (2011). **m**ADAFs (U.S. EPA 2005). **n**ASFs (OEHHA 2009).												

Recalculating LOCs, including the factors omitted by the FDA, resulted in significantly lower numbers (Table 2). Most notably, the revised LOCs for naphthalene in shellfish using the pregnant woman scenario are four orders of magnitude smaller than the FDA values (FDA 2010a). At the LOCs set by the FDA, we calculated cancer risks of 4,094 and 20,214 per million people for a combined high-shellfish diet for the woman and pregnant woman scenarios, respectively (Table 3). Although the combined high-shellfish diet scenario represents the sum of individual shellfish consumption rates, it is consistent with estimates of high-end shellfish consumption [see Supplemental Material, p. 4 (http://dx.doi.org/10.1289/ehp.1103695)]. These risks greatly exceed the FDA risk threshold of 1 in 100,000 (or 10 in 1 million) and indicate that the FDA LOCs are too high to be protective of vulnerable subpopulations.

**Table 2:** Comparison of FDA published LOCs for PAHs in Gulf seafood and revised LOCs calculated for vulnerable populations.

Vulnerable populations, revised LOCs (ppb)				
PAH, seafood type	FDA LOCs (ppb)	Woman*a*	Child*b*	Pregnant woman**(prenatal exposure)*c*
BaP								
Fish		35		0.41		0.10		0.06
Shrimp		132		1.46		0.35		0.17
Crab		132		3.05		0.75		0.36
Oysters		143		3.56		1.06		0.63
Total shellfish*d*				0.77		0.20		0.10
Naphthalene								
Fish		32,700		25.16		6.07		3.76
Shrimp		123,000		88.64		21.33		10.24
Crab		123,000		185.71		45.67		21.96
Oysters		133,000		216.67		64.48		38.31
Total shellfish*d*				46.99		11.86		5.91
**a**Adult (FDA equation): LOC = (risk level × body weight × averaging time × unit conversion factor) ÷ (cancer slope factor × consumption rate × exposure duration). **b**Child scenario (OEHHA equation): LOC = risk level ÷ {cancer slope factor × [(ADAF_2–5_ × duration_2–5 _× consumption_2–5_) + (ADAF_6–12_ × duration_6–12_ × consumption_6–12_)]}. **c**Pregnant woman scenario (OEHHA equation): LOC = risk level ÷ {cancer slope factor × [(ASF_prenatal_ × duration _prenatal_ × consumption_prenatal_) + (ASF_0–2_ × duration_0–2_ × consumption_0–2_) + (ASF_2–5_ × duration_2–5_ × consumption_2–5_) + (ASF_6 to < 10_ × duration_6 to < 10_ × consumption_6 to < 10_)]}. **d**Values reflect LOCs calculated assuming combined high-end consumption of shrimp, crab, and oysters [see Supplemental Material (http://dx.doi.org/10.1289/ehp.1103695)].								

**Table 3:** Cancer risks (excess risk per million people) calculated for vulnerable Gulf Coast populations at the LOCs set by the FDA for the Gulf Coast after the BP oil spill.

Scenario	Contaminant	Fish	Shrimp	Crab	Oysters	Total shellfish
Woman		BaP equivalents		85		968		462		40		1,470
		Naphthalene		1,300		1,388		622		614		2,624
		Total		1,385		2,356		1,084		654		4,094
Child		BaP equivalents		351		376		176		13		565
		Naphthalene		5,389		5,767		2,639		206		8,612
		Total		5,740		6,143		2,815		219		9,177
Pregnant woman		BaP equivalents		567		784		366		87		1,237
		Naphthalene		8,703		12,015		5,602		1,360		18,977
		Total		9,270		12,799		5,968		1,447		20,214


**Table 4:** Calculated cancer risks (excess risk per million people) based on mean (95% CI) detected PAH levels*a* in Gulf shellfish tested after the BP oil spill.

Scenario	BaP equivalent	Naphthalene	Total
Woman		0.008 (0.006, 0.012)		0.5 (0.1, 0.8)		0.508 (0.106, 0.812)
Child		2.1 (1.5, 3.0)		1.9 (0.5, 3.3)		4.0 (2.0, 6.3)
Pregnant woman		4.2 (3.2, 6.2)		3.9 (1.1, 6.7)		8.1 (4.3, 12.9)
**a**Combined FDA and NOAA testing using the HPLC-fluorescence method.						

*Health risks associated with Gulf Coast shellfish tested after the oil spill.* Although the volume of testing was low, government monitoring of PAH levels in Gulf seafood enables a rough calculation of the cancer risk associated with measured levels of PAHs in Gulf shellfish for populations of concern. The FDA based the reopening of coastal (state) waters to commercial shellfish harvesting on a total of 80, 37, and 92 samples of shrimp, oysters, and crab, respectively (FDA 2011). The NOAA analyzed an additional 122 shrimp samples before reopening offshore (federal) waters (NOAA 2011). Subsequently, both the FDA and NOAA have conducted follow-up testing of seafood collected in reopened Gulf waters for shrimp (*n* = 155), crab (*n* = 34), and oysters (*n* = 3).

The NOAA initially used gas chromatography-mass spectrophotometry (GC/MS) with low detection limits, but the alkyl naphthalenes were omitted, thereby underestimating total naphthalene concentrations. Subsequent NOAA testing and all FDA testing used a more-rapid high-performance liquid chromatography method (HPLC) with fluorescence detection, with a higher detection limit. We analyzed the data published on the FDA and NOAA web sites as of 10 June 2011 (FDA 2011; NOAA 2011). In addition, the Natural Resources Defense Council conducted a shrimp-sampling project in Barataria Bay, Louisiana, and the Mississippi Sound near Pass Christian, Mississippi, in December 2010 using the GC/MS analytical method, but including alkyl naphthalenes. Our project, although covering only two specific locations of concern, collected 4–9 samples per 100-mile^2^ sampling grid, greatly exceeding the sampling density the FDA reported for state waters.

We used the revised risk assessment methods to evaluate the levels of carcinogenic PAHs detected in shellfish after the oil spill. For the seven PAHs with established toxicity equivalents, we calculated total BaP equivalents to enable comparison with the LOC and to calculate total cancer risk. Detection frequencies and concentrations of carcinogenic PAHs varied between the analytes, types of shellfish, testing methods, and agency data sets [see Supplemental Material, Table 2 (http://dx.doi.org/10.1289/ehp.1103695)]. To calculate cancer risk at the levels detected in Gulf shellfish, we combined results generated using comparable analytical methods (FDA and NOAA data sets). To evaluate a worst-case scenario, and in light of high analytical limits of detection, we calculated cancer risks based on detected values and 10-year exposure duration. (See Supplemental Material for more information on our data analysis methods.)

Based on the mean of the detected PAH concentrations in shellfish, cancer risks for the woman and pregnancy scenarios were 0.008 and 4.2 in 1 million, respectively, from total BaP equivalent concentrations and 0.5 and 3.9 in 1 million from naphthalene. Combined cancer risk from all PAHs, including naphthalene, was highest for the pregnancy scenario at 8.1 [95% confidence interval (CI): 4.3, 12.9] in 1 million (Table 4). When we compared measured PAH levels (using the HPLC method) with the revised LOCs in shellfish using the pregnancy scenario, we found that 0–27% exceeded the revised LOCs set for consumption of one type of shellfish (shrimp, crab, or oysters) and 17–55% exceeded the LOC for consumption of combined shellfish types. In contrast, a much smaller number of shrimp samples (2–8%) had PAH concentrations that exceeded the revised cumulative exposure LOCs for the adult woman scenario [see Supplemental Material, Table 3 (http://dx.doi.org/10.1289/ehp.1103695)]. Levels of naphthalene and BaP equivalents measured in our pilot shrimp-sampling project were lower than values reported using the HPLC method in the FDA and NOAA data sets, and only 1 of 13 samples exceeded any of the relevant LOCs (see Supplemental Material, Table 3). Notably, using revised risk calculations and the pregnancy scenario, the revised LOCs for BaP in shrimp and total shellfish are below the limit of detection for BaP using the HPLC method (0.39 ppb). The LOC for naphthalene in shrimp for the pregnant woman scenario is below the limit of quantification of the HPLC method (15.0 ppb) (FDA 2010b).

Taken together, these findings demonstrate that the FDA’s conclusion that there were no risks to Gulf populations from oil spill–related contaminants in seafood missed some exposures of concern, particularly for pregnant women who are high-end seafood consumers. Additionally, the use of the HPLC-fluorescence analytical method, although improving the speed of analysis, may have missed low levels of PAH contamination of public health relevance for vulnerable populations.

## Conclusions

Environmental risk assessment requires the use of scientifically founded assumptions and appropriate default estimates about the exposed population, the intensity and duration of exposure, and the dose–response relationship. The risk assessment methods used by the FDA to set safe exposure levels for Gulf Coast seafood after the oil spill do not incorporate current best practices and do not protect vulnerable populations. The FDA’s conclusions about risks from Gulf seafood should be interpreted with caution in coastal populations with higher rates of seafood consumption and in vulnerable populations such as children, small adults, and pregnant women. Our analysis demonstrates that a revised approach, using standard risk assessment methods, results in significantly lower acceptable levels of PAHs in seafood and identifies populations that could be at risk from contaminants in Gulf Coast seafood. Health advisories targeted at high-end consumers would better protect vulnerable populations such as pregnant women, women who may become pregnant, and children. Our approach did not address infant exposure to PAHs via maternal seafood consumption and lactational transfer. The NRC (2008) found up to 50-fold interindividual variability in cancer risk and recommends incorporation of estimates of uncertainty, as well as population risk distributions, into future risk assessments. Improved public health protection from contaminants in food will require reforming FDA risk assessment practices.

## Supplemental Material

(238 KB) PDF
