# Supplementary material for: Seafood Contamination after the BP Gulf Oil Spill and Risks to Vulnerable Populations: A Critique of the FDA Risk Assessment
Source: Environ Health Perspect. 2011 Oct 12;120(2):157–61. doi: 10.1289/ehp.1103695 (PMC3279436; doi:10.1289/ehp.1103695)
Supplement: (238 KB) PDF [file ehp.1103695.s001.pdf]

Supplemental Material

Seafood Contamination after the BP Gulf Oil Spill and Risks to Vulnerable Populations: A Critique of the FDA Risk Assessment

Miriam Rotkin-Ellman, Karen K. Wong, Gina M. Solomon

Table of Contents

Supplemental Methods……………………………………………………………2

References…………………………………………………………………………7

Supplemental Material Table 1…………………………………………………9

Supplemental Material Table 2…………………………………………………10

Supplemental Material Table 3…………………………………………………11

Some values in Supplemental Material, Table 1, were incorrect in the Supplemental Material originally published online. They have been corrected here. The changes do not affect any of the results presented in the article.

Supplemental Methods

<u>Revised Risk Assessment and Levels of Concern</u>

General Parameters

We applied more health-protective variables used in other historical FDA oil spill seafood risk assessments to describe the acceptable risk level (1 in a million cancer risk) and exposure duration (10 years).

Adult Variables

The FDA risk assessment performed for dioctyl sodium sulfosuccinate, a component of the chemical dispersants, used a more protective body weight for adults, including women, of 60kg (Bolger 2010). To better account for high-end seafood consumption patterns on the Gulf Coast, we used consumption rates that more closely approximate high end consumers. Where available (fish, shrimp, and crab), we calculated the 95th percentile consumption rates based on the total seafood consumption rates and fraction apportioned to the individual seafood categories reported in *Seafood Choices: Balancing Benefits and Risks* (IOM 2007). We found very limited data on oyster consumption rates, particularly for high end consumers. For this analysis, we relied on the consumption frequency data collected in a study of seafood consumption rates among Louisiana Recreational Anglers (Lincoln et al. 2011) coupled with a "standard" seafood portion size from the EPA Exposure Factors Handbook. While this study is regionally specific to the Gulf Coast, the respondents were overwhelmingly Caucasian; consumption rates from other coastal communities, such as the Vietnamese fishing community, which has been identified by the US EPA as a population with significantly elevated seafood consumption rates, are likely to be significantly higher.

Early Life Exposure Adjustment Parameters

To account for the increased vulnerability of subpopulations, we evaluated two early life exposure scenarios – Child (exposure between 2-12 years old) and Pregnant woman (exposure during the 3rd trimester of gestation and continuing for 10 years). We evaluated cancer risks using US EPA and California EPA published methods for childhood and prenatal exposures (US EPA 2005, OEHHA 2009). This includes age-specific doses based on available data on consumption rates and body weight and early-life vulnerability factors. For the child scenario, we used the US EPA Age Dependent Adjustment Factors (ADAFs) and for the pregnant woman scenario, we used the California EPA's Office of Environmental Health Hazard Assessment (OEHHA) median Age Sensitivity Factors (ASFs). Subsequent to publication of the median ASFs, OEHHA utilized an ASF of 3 for the juvenile age group (2 to <16) in a risk assessment for the PAH, benzo(a)pyrene. We incorporated this information by using an ASF of 3 for the later portion of the juvenile age range (6 to <12) while maintaining the ASF 5 for the younger, more vulnerable, (2 to 5) age range. Dividing this age grouping also allowed us to apply more age-specific and relevant exposure variables.

For fish, shrimp, and crabs, we were able to use the consumption rates beginning at 2 years of age from the IOM (2007) report. The US EPA (2008) Child Specific Exposure Factors Handbook reported fish consumption rates for high-end consumers for the 0 to 2 year old interval. To approximate consumption rates for shellfish (shrimp, crabs, and oysters) during this period, we applied a scaling factor based on the consumption distribution for fish (the ratio of consumption rates for the 2-5 compared to 0-2 age groupings) to the 2-5 year old consumption rates for shellfish. Similar to adults, limited information on childhood consumption rates for oysters is available. For this assessment, we used the reported values for Native American Children in the US EPA (2011) 2011 Edition of the Exposure Factors Handbook. Prenatal exposures were

estimated as 10% of the maternal dose based on available data from animal studies on PAH toxicity (Perera et al 2005).

Total Shellfish Consumption Risk Profile

It would be expected that high-end seafood consumers are not just eating one type of shellfish; therefore cancer risks should reflect the total shellfish diet. We calculated revised LOCs to reflect this combined shellfish consumption by apportioning the acceptable risk according to the relative fraction of the combined intake of our estimated individual shellfish consumption rates. Although this is a high end value (83g/day), it falls within the range reported for the 99th percentile of national total shellfish consumption for adults in the EPA (2011) Exposure Factors Handbook and is below the 95th percentile value reported for consumers in Florida. In the absence of regionally specific total shellfish consumption rates for children and adults, our combined intake gives a reasonable estimation of high-end exposures to contaminants in shellfish.

<u>LOC calculations</u>

For the adult/woman LOC calculation we used FDA's equation:

LOC (BaP equivalent) = (Risk Level * Body Weight * Averaging Time * Unit Conversion Factor) / (Cancer Slope Factor * Consumption Rate * Exposure Duration)

For the child and pregnant woman scenarios we derived an equation similar to that used by OEHHA to calculate a drinking water standard for hexavalent chromium (OEHHA 2011):

Child scenario LOC = Risk Level / Cancer Slope Factor * $\{(ADAF_{2\text{-}5}*duration_{2\text{-}5}*consumption_{2\text{-}5}) + (ADAF_{6\text{-}12}*duration_{6\text{-}12}*consumption_{6\text{-}12})\}$

Pregnant woman scenario LOC = Risk Level / Cancer Slope Factor * $\{(ASF_{prenatal}*duration_{prenatal}*consumption_{prenatal}) + (ASF_{0\text{-}2}*duration_{0\text{-}2}*consumption_{0\text{-}2}) + (ASF_{2\text{-}5}*duration_{2\text{-}5}*consumption_{2\text{-}5}) + (ASF_{6\text{-}<10}*duration_{6\text{-}<10}*consumption_{6\text{-}<10})\}$

The majority of these variables are described in Table 1 in the main text. Additional variables include:

Averaging Time = 78 years

Cancer Slope Factor = benzo(a)pyrene:7.3 $(mg/kg\text{-}day)^{-1}$ and naphthalene 0.12 $(mg/kg\text{-}day)^{-1}$

Unit Conversion Factor = 1,000g/kg

Duration = age-specific exposure period/averaging time (78 years)

Consumption = consumption rates expressed as kg – seafood/kg-body weight per day

Health risks associated with Gulf Coast shellfish tested after the oil spill

We analyzed seafood testing data collected by the FDA and NOAA to determine health risks associated with the reported carcinogenic PAH levels. During the first round of testing, NOAA utilized a different analytical method from FDA and from later sampling events. Therefore, the results are not comparable and are reported separately. Results reported by FDA included detected values, the designation of TR for "trace," and non-detect. In this analysis, we evaluated all detected levels of carcinogenic PAHs. To accomplish this, we assigned the samples marked "trace" with a value equal to half the level of quantification reported for that analyte-seafood

type pairing in the FDA's Laboratory Information Bulletin for the analytical method (US FDA 2010b). For each type of seafood, PAH, and responsible agency, we calculated the total number of samples available (from oil-spill impacted areas), the number and percent of samples with detectable levels of PAHs, the mean and 95% Confidence Interval (CI) of the detected values, and the percent of samples that exceeded the revised LOCs (Supplemental Tables 2&3). The 7 PAHs for which established cancer toxicity equivalents have been derived can be summed using the cancer equivalent values to reflect their relative potency as compared to benzo(a)pyrene. We used this method to calculate total BaP-equivalents and compared this value to the revised LOCs. We also calculated estimated excess cancer risk for our 3 Gulf Coast Vulnerable Populations scenarios associated with the mean (and 95th CI) values of total BaP-equivalents and naphthalene (Table 4). For this calculation, we averaged the available data for shrimp (FDA and NOAA HPLC analytical method data) and also averaged the concentrations of PAHs in crab meat and crab hepatopancreas. We assumed that a high end consumer is eating all three different types of seafood and therefore calculated cancer risks from combined shellfish exposures and for total cancer risk from BaPe and naphthalene exposures.

References

Bolger M. 2010. Levels of Concern (LOC)s for Select Gulf Seafood Species. Memorandum from M Bolger, Chemical Hazards Assessment Staff, Office of Food Safety, Food and Drug Administration to D Kraemer Center for Food Safety and Applied Nutrition. Available: http://www.fda.gov/downloads/Food/FoodSafety/Product-SpecificInformation/Seafood/UCM231697.pdf. [Accessed 20 June 2011]

FDA (Food and Drug Administration). 2010b. Laboratory Information Bulletin: Screen for the Presence of Polycyclic Aromatic Hydrocarbons in Select Seafoods using LC-Fluoresence.

Law RJ, Hellou J. 2002. Contamination of Fish and Shellfish following Oil Spill Incidents. Environ Geosci. 1999;6(2): 90-98.

Lincoln RA, Shine JP, Chesney EJ, Vorhees DJ, Grandjean P, Senn DB. 2011. Fish consumption and mercury exposure among Louisiana recreational anglers. Environ Health Perspect. 119:245-251.

Institute of Medicine 2007. Seafood Choices: Balancing Benefits and Risks. (Nesheim MC, Yaktine AL, eds) Washington DC: The National Academies Press.

OEHHA (Office of Environmental Health Hazard Assessment). 2009. In utero and early life susceptibility to carcinogens: The derivation of age-at-exposure sensitivity measures. California: California Environmental Protection Agency.

OEHHA (Office of Environmental Health Hazard Assessment). 2011. Public Health Goals for Chemicals in Drinking Water Hexavalent Chromium (Cr VI): California: California Environmental Protection Agency.

Perera F, D Tang, Whyatt R, Lederman SA, Jedrychowski W. 2005. DNA Damage from Polycyclic Aromatic Hydrocarbons Measured by Benzo[a]pyrene-DNA Adducts in Mothers

and Newborns from Northern Manhattan, The World Trade Center Area, Poland, and China. Cancer Epidemiol Biomarkers & Prev. 14:709-714.

US EPA (U.S. Environmental Protection Agency). 2005. Supplemental guidance for assessing susceptibility from early-life exposure to carcinogens. EPA/630/R-03/003F.

US EPA (U.S. Environmental Protection Agency). 2008. Child Specific Exposure Factors (CSEF) Handbook.

US EPA (U.S. Environmental Protection Agency). 2011. Exposure Factors Handbook: 2011 Edition. EPA/600/R-090/0S2F

Supplemental Material, Table 1: Revised LOCs for other (non – naphthalene) carcinogenic PAHs (ppb) calculated based on the Benzo(a)pyrene equivalent (BaPe) LOC and the established cancer toxicity equivalents.

		BaPe	Benzo (a) pyrene	Dibenz(a,h) anthracene	Indeno(1,2,3-cd)pyrene	Benz(a) anthracence	Benzo(b) fluoranthene	Benzo(k) fluoranthene	Chrysene
	Cancer equivalents		1	1	0.1	0.1	0.1	0.01	0.001
Fish	woman	0.41	0.41	0.41	4.10	4.10	4.10	41.00	410.00
	child	0.10	0.10	0.10	1.00	1.00	1.00	10.00	100.00
	pregnant woman	0.06	0.06	0.06	0.60	0.60	0.60	6.00	60.00
Shrimp	woman	1.46	1.46	1.46	14.60	14.60	14.60	146.00	1460.00
	child	0.35	0.35	0.35	3.50	3.50	3.50	35.00	350.00
	pregnant woman	0.17	0.17	0.17	1.70	1.70	1.70	17.00	170.00
Crab	woman	3.05	3.05	3.05	30.50	30.50	30.50	305.00	3050.00
	child	0.75	0.75	0.75	7.50	7.50	7.50	75.00	750.00
	pregnant woman	0.36	0.36	0.36	3.60	3.60	3.60	36.00	360.00
Oyster	woman	3.56	3.56	3.56	35.60	35.60	35.60	356.00	3560.00
	child	1.06	1.06	1.06	10.60	10.60	10.60	106.00	1060.00
	pregnant woman	0.63	0.63	0.63	6.30	6.30	6.30	63.00	630.00
Total Shellfish	woman	0.77	0.77	0.77	7.70	7.70	7.70	77.00	770.00
	child	0.20	0.20	0.20	2.00	2.00	2.00	20.00	200.00
	pregnant woman	0.10	0.10	0.10	1.00	1.00	1.00	10.00	100.00

Supplemental Material, Table 2: PAH levels of Gulf shellfish tested after the Oil Spill

			BaP equivalent (BaPe)			Naphthalene		
			Number (%) Detected [b]	detected values (ppb)		Number (%) Detected [b]	detected values (ppb)	
Seafood Type	Data source [a] (analytical method)	Total samples		mean	95% CI		mean	95% CI
Shrimp	FDA - (HPLC)	136	9 (7%)	0.4	(0.1-0.8)	37(27%)	18.1	(2.7-33.5)
Shrimp	NOAA - (GC/MS)[c]	121	44(36%)	5.00E-04	(4E-4 - 6E-4)	121(100%)	0.93	(0.86-1.00)
Shrimp	NOAA - (HPLC)	99	4(4%)	0.7	(0.0-1.5)	76 (77%)	8.9	(7.9-9.9)
Shrimp	NRDC - (GC/MS)[c]	13	0	N/A	N/A	5(38%)	2.72	(0.13-5.32)
Oyster	FDA -(HPLC)	40	12(30%)	0.27	(0.06-0.47)	17(43%)	5.9	(4.2-7.7)
Crab meat	FDA -(HPLC)	77	9 (11%)	0.2	0.12-0.27)	8(10%)	21.3	(14-28.7)
Crab Hepatopancreas	FDA -(HPLC)	38	20 (53%)	0.18	(0.16-0.20)	3(8%)	86	(7.1-146.9)

[a] FDA testing was conducted from August 2010 through February 2011, NOAA testing was conducted from August 2010 through January 2011 and NRDC testing was conducted in December 2010.

[b] The limit of detection (LOD) for the HPLC method varied by PAH and seafood type; shrimp (0.23 -8.2 ppb), oyster (0.39 – 7.3 ppb), crab (0.33 – 20 ppb). According to NOAA shrimp data reports, the LODs for the GC/MS method ranged from 0.12 to 0.25 ppb. The LODs for the NRDC sampling ranged from 0.2 to 1.0 ppb.

[c] NOAA GC/MS testing does not includes alkyl homologues; in the NRDC testing, alkyl homologues were analyzed separately and summed.

[d] Background data for PAH levels in the Gulf of Mexico shellfish are unavailable and it is difficult to generalize based on the available literature due to variations in testing methods and composition (i.e. varying suites of PAHs are tested for) of the analysis. Law et al (2002) attempted to standardize the literature using the BaPequivalent approach and reported a range of 0 to 222 ppb BaPe from the US mussel watch monitoring program 1986-1996 data files.

Supplemental Material, Table 3: Percent of Gulf seafood samples tested after the BP oil spill that exceed revised LOCs

Seafood Type	Data source (analytical method)	Total samples	Percent of Samples Exceeding Revised LOCs								
			Seafood and Contaminant Specific LOCs						Cumulative exposure LOCs		
			Woman		Child		Pregnant Woman		Woman	Child	Pregnant Woman
			BaPe	Naph-thalene	BaPe	Naph-thalene	BaPe	Naph-thalene	Combined*		
Shrimp	FDA - (HPLC)	136	0%	1%	2%	3%	4%	4%	3%	7%	26%
Shrimp	NOAA - (GC/MS)	121	0%	0%	0%	0%	0%	0%	0%	0%	0%
Shrimp	NOAA - (HPLC)	99	0%	0%	2%	1%	2%	27%	2%	24%	53%
Shrimp	NRDC (GC/MS)	13	0%	0%	0%	0%	0%	0%	0%	0%	8%
Oyster	FDA -(HPLC)	40	0%	0%	0%	0%	10%	0%	8%	18%	28%
Crab meat	FDA -(HPLC)	77	0%	0%	0%	0%	0%	6%	0%	12%	17%
Crab Hepatopancreas	FDA -(HPLC)	38	0%	0%	0%	0%	0%	13%	5%	16%	55%

* Concentrations of carcinogenic PAHs (including naphthalene) were compared to LOCs revised to incorporate total shellfish consumption. As per FDA procedures for carcinogenic PAHs, samples were flagged where the combined ratio of the concentration to the relevant LOC exceed one.
